# The Use of Digital Technology to Support the Emotional Well-Being of Older People Who Receive Care at Home From the Perspective of Care Recipients: Qualitative Study

**DOI:** 10.2196/93823

**Published:** 2026-10-28

**Authors:** Tanya E Davison, Hilary Davis, Marissa Dickins, Sonja Pedell, Tuan Anh Nguyen, Deborah Carveth, Therese Den Dulk, Karen Smith, Julie Kelly, Sunil Bhar

**Affiliations:** 1 Research and Innovation Silver Chain Group Ltd Melbourne, Victoria Australia; 2 Department of Design Faculty of Art, Architecture and Design Monash University Melbourne, Victoria Australia; 3 Department of Psychological Sciences School of Health Sciences Swinburne University of Technology Melbourne, Victoria Australia; 4 Centre for Social Impact School of Business, Law and Entrepreneurship Swinburne University of Technology Melbourne, Victoria Australia; 5 School of Public Health and Preventive Medicine Faculty of Medicine, Nursing and Health Sciences Monash University Melbourne, Victoria Australia; 6 Centre for Design Innovation School of Design and Architecture Swinburne University of Technology Melbourne, Victoria Australia; 7 Centre for Mental Health and Brain Sciences School of Health Sciences Swinburne University of Technology Melbourne, Victoria Australia; 8 Social Gerontology Division National Ageing Research Institute Melbourne, Victoria Australia; 9 College of Health Sciences VinUniversity Hanoi Vietnam; 10 School of Pharmacy and Biomedical Science Adelaide University Adelaide, South Australia Australia

**Keywords:** aged care, home care, social care, digital technology, older adults, well-being, mental health, digital health, digital literacy, digital usability

## Abstract

**Background:**

Older people who receive aged care services at home experience high rates of depression, anxiety, and loneliness, yet have poor access to services to support their well-being. Digital interventions are widely available, but research is required to determine whether they are acceptable to the in-home aged care population.

**Objective:**

This study aimed to explore the perspectives of in-home aged care recipients on the use of digital technologies to support their emotional well-being.

**Methods:**

Eleven older people (aged 70-87 years) who received in-home aged care from a national provider participated in a semistructured interview. The interview aimed to understand care recipients’ experiences of using technology, as well as their current access to emotional support and attitudes toward using digital technologies to support their well-being.

**Results:**

Participants reported high access to technology (mean 5, SD 1.6 devices) but varied usage. Most required support to use technology, typically provided by family members, and reported a range of concerns related to technology use. Participants received a variety of informal supports for their emotional well-being, typically from family members and their communities, but only one accessed professional support. Most were open to using technology to support their emotional well-being, and some were willing to receive such support from their aged care provider.

**Conclusions:**

The willingness of older people who receive in-home aged care to use digital technologies to supplement existing informal emotional support is encouraging. Aged care providers are well placed to implement digital well-being programs and can leverage older people’s existing access to digital devices but should consider their requirements for technical support. Technologies and implementation strategies should be purpose-designed for this population and address identified barriers.

## Introduction

Globally, population aging has resulted in increased demand for health care and aged care services, with growing numbers of older people requiring assistance to manage increasingly complex needs [1]. Older people typically prefer to remain at home for as long as possible [2], and many countries have observed significant increases in the number accessing in-home aged care (also known as social care). For example, in Australia, the number of older people who received a government-funded Home Care Package to enable them to remain at home nearly quadrupled over the past 10 years [3].

Symptoms of depression and anxiety, and other indicators of poor emotional well-being, such as loneliness, social isolation, grief, and adjustment difficulties, affect more than half of older people who receive aged care at home [4,5] and appear to be increasing alongside other chronic health conditions, frailty, and multimorbidity [6]. Aged care providers commonly provide social support to lonely or socially isolated clients (eg, drive clients to social events or provide company in the home) and may escalate to the older person’s medical practitioner for follow-up if they suspect a mental health problem. However, aged care recipients have limited access to specialist services, with recent research indicating that only 2.5% of home care recipients in Australia use government-funded mental health support [7].

With shortages in both aged care and mental health workforces [8,9], alternative approaches are required to address the growing mental health and well-being needs of aged care recipients. Older adults experience significant barriers to accessing outpatient services, especially those with impaired mobility and who live in regions where services are sparse [10]. Digital technologies offer an opportunity to deliver interventions and support at scale to reach large numbers of older people with depression, anxiety, and other aspects of poor emotional well-being who currently have poor access to support and address the current scarcity of mental health in-reach programs in the community [11].

There has been enormous growth in the development of digital mental health and well-being interventions in recent years. These interventions use a variety of technologies and can be grouped into several overlapping categories based on existing taxonomies [12]: (1) synchronous provider-delivered support, such as counseling or psychotherapy delivered by videoconference; (2) guided digital interventions, including web-based or app-based programs supported by a clinician, coach, or facilitator; (3) self-guided tools, such as mobile apps or websites for mood monitoring, psychoeducation, mindfulness, relaxation, or activity planning; (4) social connection technologies, including video calling, online peer-support groups, forums, and social media platforms; and (5) supportive or assistive technologies, such as smart speakers, reminders, and conversational agents. This typology highlights that digital support for emotional well-being extends beyond formal treatment of depression or anxiety to include technologies that foster social connectedness, meaningful activity, and day-to-day self-management in the home environment. The paper uses “emotional well-being” as an umbrella term to encompass mental health symptoms and conditions, as well as related aspects of well-being, including distress, grief, sense of purpose, and social connection. This usage is consistent with recent conceptualizations of emotional well-being as encompassing affective experience, meaning and purpose, and social connection [13].

While most technologies to support emotional well-being have been designed for younger people [14], there is promising evidence that some digital interventions (eg, online cognitive behavior therapy) are effective in reducing symptoms of depression in older people when designed specifically for this population [15], although reviewers have noted that more research is needed [16]. Digital interventions may also be effective in improving loneliness and social connection in older people [17,18]. However, there is little work exploring their use in older people who receive aged care at home.

Older people are generally open to learning to use digital devices [19], and there is an established literature on the use of digital technologies to facilitate health care by older people [20]. Research is also emerging about the views and experiences of older people in using digital technologies to support their emotional well-being, which has been synthesized in recent literature reviews [11,19]. While digital technologies offer convenience and anonymity [19], identified barriers for older people include doubts about the usefulness of digital approaches for emotional well-being, poor access to technology and low digital literacy, as well as physical, cognitive, or sensory impairments, and concerns related to privacy and security [11,19]. Older people with mental health disorders such as depression [19] appear more hesitant to use digital interventions and overall, older people may prioritize the management of other aspects of their health, such as chronic illness, pain, and medications, over mental health and emotional well-being [11]. These findings indicate it is important to consider attitudes toward the use of digital interventions within the broader context of older people’s willingness to attend to mental health issues and access emotional support more generally.

There is a particular gap in research examining the use of technology to support emotional well-being among older people who require aged care services to remain at home. Aged care recipients are significantly older than the “older people” recruited to most research studies (eg, in Australia, the median age at commencement of home care services is 81 years [3]) and this population experiences high rates of multimorbidity and frailty [6], as well as other indicators of complexity, such as cognitive decline [21]. While there is growing adoption of technology by older people generally, the oldest old and those in poor health are more likely to experience a digital divide [22]. Thus, research on use, facilitators, and barriers in relation to digital interventions for emotional well-being derived from general older adult community samples may not generalize to this group. There are indications from interviews with aged care providers that some care recipients are willing to use technology to maintain their social connections, with providers reporting strong uptake of digital communication technologies among their clients during the COVID-19 pandemic [10]. However, there is a lack of published literature on the perspectives of in-home aged care recipients themselves regarding the use of technology to support their emotional well-being.

This study had 3 aims. First, we aimed to gather information from older adults accessing in-home aged care about their experience in using digital technologies in general. Second, we aimed to explore their current access to emotional support and their views on using technology to support their emotional well-being. Finally, we aimed to understand care recipients’ willingness to take up such programs when offered by the organization providing their aged care services.

## Methods

### Study Design and Setting

An interview study was conducted with older people receiving aged care services at home from a single provider. The service provider offers a range of aged care services, including domestic support, personal care, social support, home modifications, nursing, allied health, and transport.

### Participants

Study participants were in-home aged care clients who resided in metropolitan Victoria, Australia. Clients were approached by their aged care provider if they were considered to have poor emotional well-being, such as depression or low mood, based on their professional judgment, and were potentially interested in participating in a study on how technology can improve older people’s mood and emotional well-being. Participants were not required to have a diagnosis of a mental health disorder, and no mental health or well-being screening was conducted for this study. Eligibility criteria included age of 65 years or older and current receipt of a government-funded aged care service. Participants were not required to have any level of expertise in technology or digital literacy. Exclusion criteria included a diagnosis of dementia or presence of significant cognitive impairment, an acute health-related issue that could interfere with research participation, and difficulty engaging in an interview conducted in English.

### Procedure

After an initial conversation with the aged care provider, if the client indicated an interest in taking part in the research, a researcher called them on the telephone, confirmed their willingness to participate, and set up a time to visit their home for the interview. The participant information sheet and consent form were mailed or emailed to all participants prior to the interview. A researcher visited the client’s home, obtained written consent, and conducted the audio-recorded interview, using a semistructured interview format. Participants were given the opportunity to have a family carer present during the interview.

### Materials

Demographic variables included client age, sex, living arrangement, and geographical location, which were obtained from the participant’s care record prior to the home visit. Additional data were collected during the visit: level of education, marital status, and a single item on the presence or absence of a health problem that prevents the participant from doing things people their age can normally do, which was answered “Yes,” “No,” “Don’t Know,” or “Prefer Not to Disclose.” A full list of demographic questions is available in Multimedia Appendix 1.

A semistructured interview was developed for the study, with interview questions exploring care recipients’ existing access to and use of technology, current access to support for their emotional well-being, and their views on the use of digital technologies to improve their mood or emotional well-being, such as web-based platforms, mobile apps, or videoconferences. Questions also explored their views of emotional support being offered by their aged care provider (refer to Multimedia Appendix 1 for interview schedule).

### Analysis

Demographic data were analyzed using descriptives (eg, mean, range, and frequency). Qualitative data were transcribed and coded using thematic analysis: a “method for identifying, analyzing and reporting patterns (themes) within data” to find “repeated patterns of meaning” [23]. The 6 phases of thematic analysis described by Braun and Clarke [23], namely, familiarization, generation of initial codes, searching for themes, reviewing themes, and naming themes, followed by report production, were used. No formal codebook was developed. Instead, an inductive strategy was used, which allowed the themes to arise from the data, and the phases of analysis were treated in a reciprocal manner, allowing movement back and forth between the phases. All transcripts were coded by 3 team members (DC, TDD, and HD) using Microsoft Excel. Any discrepancies were resolved through consensus during discussions. Regular discussions were held within the multidisciplinary research team to provide further insight into the themes arising from the analysis. Quotes were selected based on their ability to capture the theme accurately and articulately, ensuring that perspectives were drawn from across the participants.

### Ethical Considerations

Ethical approval was granted by the Swinburne University of Technology Human Research Ethics Committee, and data were collected between October and December 2023.

## Results

### Participant Characteristics

Fifteen clients were nominated for the study by the aged care provider. Two did not meet eligibility criteria (one due to dementia and one due to language issues), 1 could not be contacted, and 1 declined to participate. The final client sample included 11 aged care recipients (7 female participants and 4 male participants), who were all retired and ranged in age from 70 to 87 (mean 79, SD 5.2) years (Table 1). The sample size of 11 was determined to be sufficient as interviewers identified that saturation had been reached, with no new themes arising in the final interview, and data collection was ceased. Seven participants lived alone, while 4 lived with family members, and 7 were born in Australia. There was a range of formal education: most did not finish secondary school (n=7), but 1 participant had completed secondary schooling, 2 had completed technical or vocational training, and 1 had completed an undergraduate degree. Participants were either married (n=5), widowed (n=4), or divorced or separated (n=2). The majority were living with a health problem that prevented them from doing things people their age can normally do (n=7), but 2 answered “no” to this question, and 2 either were not sure or elected not to answer. Three clients were accompanied by a family carer in the interview, typically their spouse; however, in order to focus on the perspectives of clients themselves, only comments from the clients were included in this analysis.

**Table 1:** Participant characteristics and technology ownership and usage.

Participant	Sex	Age (years)	Education status	Technology owned	Technology usage
1	Female	78	2 years of secondary school	Smartphone, tablet, computer, smartwatch, and internet access	Communication (calls, SMS, WhatsApp, and Messenger), entertainment (YouTube), and social media (faith groups on Facebook)
2	Female	79	3 years of secondary school	Smartphone, tablet, computer, smart TV, and internet access	Communication (calls and SMS), entertainment (puzzles such as Sudoku), information and tasks (plans holidays using Google), and social media (Facebook: reads only, does not post) Limited use of smartphone
3	Male	83	Technical college	Smartphone, tablet, computer, smart TV, internet access, and electronic book (e-book) reader	Communication (calls, SMS, email, FaceTime, and Zoom), entertainment (YouTube, Netflix, Amazon Prime, news, and reading books), and information and tasks (internet banking, postal services, maps, monitoring electricity usage, booking appointments with doctors, and managing pension)
4	Male	78	4 years of secondary school	Smartphone, tablet, smart TV, and internet access	Communication (calls and email [can only read, not respond], video calls, and unable to use SMS), entertainment (Netflix and YouTube), information and tasks (booking appointments with doctors), and social media (Facebook but rarely posts)
5	Female	83	Vocational training	Smartphone, tablet, computer, smart TV, and internet access	Communication (calls and FaceTime), entertainment (Foxtel, Netflix, Disney+, ABC, and Solitaire, news), and information and tasks (Google)
6	Male	73	University degree	Smartphone, tablet, computers (1 running macOS and 1 running Windows), smart TV, and internet access	Communication (calls, SMS, WhatsApp, and email, video calls), information and tasks (online courses, Google, Calendar, and Photoshop), entertainment (YouTube, Netflix, news), and social media (Facebook and Twitter: reads only, does not post) Has difficulty using smartphone given size of text
7	Female	70	2 years of secondary school	Smartphone, computer, smart TV, smartwatch, and internet access	Communication (calls and SMS), entertainment (games), information and tasks (weather, calendar, Google, monitors steps and heart rate on watch, taking photos, and asks questions using Alexa or Siri), and social media (Facebook) Unable to use laptop, email, or phone banking
8	Male	86	4 years of secondary school	Smartphone, tablet, smart TV, and internet access	Communication (calls only), information and tasks (Google), and entertainment (Netflix and YouTube) Has an email account but managed by daughter Has access to social media (Facebook) but does not use it Does not use tablet
9	Female	75	Did not complete primary school	Smartphone, tablet, computer, smart TV, and internet access	Communication (calls, learning to use SMS, WhatsApp, and email [view only]), information and tasks (takes photos), and social media (Facebook but is limited in what she can do) Does not use the computer in the house, and limited use of tablet
10	Female	79	2 years of secondary school	Smartphone, computer, no internet at home, and no tablet or smart TV	Communication (calls and SMS) and information and tasks (taking and sharing photos) Does not use laptop
11	Female	87	Finished secondary school	Smartphone, tablet, computer, smart TV, smartwatch, internet access, and smart speakers	Communication (calls, SMS, and email), entertainment (Netflix, Disney+, listening to music on smart speakers, and news), and information and tasks (Google for health information)

### Technology Ownership and Use

Results are summarized in Multimedia Appendix 2, including illustrative participant quotes.

#### Technology Ownership

Participants reported owning 5 different types of technology (range 2-7) on average. Eleven participants owned a smartphone, 9 owned a tablet, 9 owned a laptop or desktop computer, 9 had a smart TV, 3 had a smartwatch, 1 had a smart speaker, and 1 had an electronic book (e-book) reader. Levels of internet access were also high (91%); only 1 participant reported no internet access at home (Table 1).

#### Technology Use

Collectively, participants reported using technology for a range of reasons, including to access general media, such as news or current affairs programs (n=4); social media (n=7); video entertainment, such as television shows, movies, or YouTube videos (n=7); online games (n=3); and for communication purposes, for example, sending and receiving emails (n=5, though with varying capabilities), SMS text messaging (n=8), and video calls (n=5; Table 1).

Participants commonly streamed television and movies on their smart TV (n=7), but typically only after being set up by family members:

My daughter and son bought me a smart TV and they organized for it all to be hooked up. I wouldn’t have a clue that they did, but I’ve watched a lot of Netflix.

Participant 4, male, 78 years

The social media platform Facebook (Meta Platforms, Inc) was also commonly viewed (n=7) but participants typically did not post themselves or provide comments:

I look at Facebook. I don’t know how to get it, to get in to put anything on it...but I do look at it. Yeah, you know, what everybody’s saying or looking at, you know.

Participant 9, female, 75 years

While 6 care recipients reported using technology to seek information from the internet, only 4 reported using technology to manage physical health issues. One person used a smartwatch to monitor steps taken and heart rate, 1 participant described searching for health advice online, and 2 reported using technologies to manage clinician appointments via app or SMS text messages.

The use of devices varied markedly between participants. Some (n=3) accessed a broad range of digital functionality across multiple devices to support their daily activities, including managing email, calendars and banking, and engaging in online forums. Within this group, 2 participants operated digital devices using a voice-activated digital assistant such as Alexa or Siri, and 1 participant used a smartwatch to take calls and messages:

[Smartwatch] has got everything. Yeah, it’s got the lot, and you can receive messages, and I can also talk on it too. If I’m out mowing or something, you know?

Participant 11, female, 87 years

Remaining participants (n=8) made more limited use of the potential functionality offered by their devices. Many only used their smartphone for calls and/or SMS text messaging (n=6), and in some cases, to play simple games (n=3) or take photographs (n=3):

I only ring my family, my husband. He rings me. And well, I send messages to the family. That’s all I use it [mobile phone] for. And if I get a photo of it, take a photo of the garden, I’ll send it to them and stuff, but that’s it.

Participant 10, female, 79 years

Several participants (n=5) described difficulty using core functions, such as email and SMS text messaging:

If I get an email, I don’t know how to respond to it...I don’t know how to send a text message, but yeah, I just, I would rather speak to somebody

Participant 4, male, 78 years

In addition, a small number of participants (n=4) reported they did not use at least one of the devices (smartphone, tablet, or computer) in their home at all, which in some cases (n=3) had been provided by their family members:

I don’t even want to know how to use things on my phone. Not interested... I just say I’m just too old to start learning things.

Participant 2, female, 79 years

#### Support in Using Technology

While participants described a wide range of technology use, most participants (n=8) reported receiving at least some support to use technology, typically from a close family member. In some cases (n=3), adult children appeared to routinely take charge of tasks managed through technology. This often involved sharing sensitive or personal information, such as passwords or banking information:

The daughter has [my banking information] on her computer because, um, like I got...a message I guess it was saying that the registration was due on the car and so I took it around and she said “there you are, done and paid for” because she can have access to my bank account.

Participant 4, male, 78 years

However, one participant indicated that they only turned to family members if they could not resolve the issue themselves:

Oh, I’d try to [do it myself], if it was something I absolutely couldn’t do...So yes, I did go to [my son-in-law] because I couldn’t work out what was going on.

Participant 11, female, 87 years

While adult children were often seen as a primary source of information and training in using technology, in some cases (n=2), participants’ continued use of technologies required ongoing support that did not eventuate, limiting participants’ ability to make full use of the technologies:

I need to be shown more than once, that’s my problem...I really just want to be able to say, “Yes, I can use the computer,” yeah. And you know, years ago, the kids taught me, and I was doing bits and pieces, but now it’s just got photos on it.

Participant 7, female, 70 years

No participants reported accessing technology support available from technology companies (eg, Apple and Microsoft) or independent organizations that provide technology support. One participant reported receiving support available in their local community to use technology:

When we first moved into the village here, they had a woman come to show us different things. That lasted for about four weeks. Once a week she would come in and show us how to use our laptop, so that’s good.

Participant 5, female, 83 years

There were, however, some participants (n=4) who were confident in using a range of devices, and described positive attitudes towards learning new technologies, and detailed technological knowledge:

Yes, I’m using two computers. One is Macintosh, one is Windows...because Apple doesn’t have all the programs.

Participant 6, male, 73 years

It’s just interesting, it’s a new challenge. Something nice, new to learn, yeah.

Participant 11, female, 87 years

#### Barriers to Technology Use

Despite technology being common in their homes, participants reported a range of barriers to technology use. Approximately half the participants (n=5) expressed concerns around privacy and scams. For example, this participant discussed low trust in using their smartphone and the high volume of spam calls they were experiencing:

Trusting...no! It’s no secret anyone can get our data...at least this is the nuisance from our telephone, coming about at least six or seven calls coming or spam, that is a headache.

Participant 6, male, 73 years

Cost was identified as a barrier to use of some technologies for 2 participants. One participant on an aged pension described having to choose between having internet access at home and other priorities, leaving them without core technological infrastructure:

I can’t afford to live and save up a bit of money to go on a holiday and have those sort of things [internet access], as well on an age pension.

Participant 10, female, 79 years

Other participants (n=2) raised concerns about breaking the technology or engaging with it in a way that could cause a negative outcome, which limited the technologies they chose to use and how they used them:

I will never do phone banking [on an app], okay? Because I’m worried that I might press the wrong button.

Participant 7, female, 70 years

If I have to learn something new...I just panic if I’m gonna do the wrong thing, you know? And I think and then I’m going to be in big trouble...And that’s my fear of it, you know.

Participant 9, female, 75 years

Several participants (n=3) reported the need to make minor modifications to their devices, such as increasing font size, to reduce usability barriers:

Yes (I use it) but I don’t like the phone because it’s too small. I can’t read and every, every time I have to make [the font] bigger, bigger and read, so it’s big.

Participant 6, male, 73 years

### Support for Emotional Well-Being

Results are summarized in Multimedia Appendix 2, including illustrative participant quotes.

#### Current Sources of Support

Participants were asked to describe any sources of support they engaged in for their emotional well-being. Only 1 participant reported not receiving emotional support from any source. Seven of the 11 participants reported they received emotional support from family members (in particular, their daughter), which was most commonly provided in person or over the phone:

My daughter rings every day. She’s just rung about half an hour ago.

Participant 5, female, 83 years

You just get lonely by yourself. And we just sort of support each other that way, you know...I’ve got 4 kids. I know I’ve always got them. But if I’ve got something on my mind, I’ll sort of talk it over with [my daughter], you know?

Participant 2, female, 79 years

Six participants reported receiving emotional support from their broader community, although the nature of this support differed, including informal support from friends and neighbors and formal support from a community center. For example, this participant described receiving support from others in their retirement village who had experience with grief:

A lot of people in the village here have all that [grief]...all gone through it, and we talk a lot to people there, about [grief and loss]

Participant 3, male, 83 years

In contrast, only 1 participant reported receiving current professional support for their emotional well-being, which was organized by her general practitioner (GP). Another participant had also accessed a counselor but several years ago:

I’ve got another 4 visits, but it’s up to the doctor, I think to ask for more if I need it. As soon as I mentioned it, he was straight on [organized a referral]. I had [a phone call] the next day.

Participant 7, female, 70 years

I used to [be depressed], but not anymore...oh, going back how many years? 1995. So, it’s been a lot of years and I, I spoke to a counselor and that and yeah, I’m fine.

Participant 10, female, 79 years

#### Barriers to Seeking Support

Remaining participants gave reasons for not seeking professional help. This was commonly expressed in terms of not wanting “to be a burden on anybody” (Participant 4, male, 78 years) or preferring to discuss personal details with their family:

I like to talk about myself with my family members, not others.

Participant 6, male, 73 years

One participant described difficulty accessing the support he wanted, citing issues with waiting times as a critical reason for discontinuing his search for support:

I must admit everything seems to just take so long, and, and you get to the point where you just say “Oh, forget it!” And you don’t carry on with it.

Participant 8, male, 86 years

#### Willingness to Engage in Support

Participants were asked if they would be willing to engage in professional help to support their well-being in the future, if available. The majority (n=10) indicated they were open to receiving support for their emotional well-being. However, participants commonly expressed initial reticence before identifying circumstances in which support could be useful:

No, I say no...But you never know because a friend of mine committed suicide. So, I say, you know, at the moment, no, I wouldn’t use it, but you never know, I mean, probably it’s a shame that he didn’t speak to somebody...Well, maybe, yeah, maybe in time. Yeah, I would say maybe probably once I give up driving. I think then yes maybe.

Participant 4, male, 78 years

There are times when you feel that you would probably like to talk to someone else, other than family...It’s never struck me as being absolutely necessary, but I think there are times when it'd be nice if it was there.

Participant 8, male, 86 years

While the majority (n=10) spoke about support in terms of speaking with a professional, 1 participant noted a preference for peer support for their emotional well-being:

I think it’d be the fact that I can talk to somebody else, a stranger who doesn’t know the history, but has maybe gone through something like that themselves, or knows of somebody. Umm, but just to get some feedback.

Participant 7, female, 70 years

Only 1 participant stated they would not consider engaging in professional emotional support:

No, no. Strangers. They’d be strangers. People I didn’t know that would be talking, probably from rote. No, no, it wouldn’t, wouldn’t mean a thing to me.

Participant 11, female, 87 years

### Use of Digital Technologies to Support Emotional Well-Being

Results are summarized in Multimedia Appendix 2, including illustrative participant quotes.

#### Current Use of Technology to Support Emotional Well-Being

Participants were asked to share their current experiences using technology to support or enhance their well-being. Seven participants reported diverse experiences, including engaging with music, hobbies such as games, quizzes, or reading, and remote religious support:

[I] read a lot, I’ve got a little Kindle [eReader], and I sit at the computer for half an hour and just see what’s happening in the world.

Participant 3, male, 83 years

On my phone, I have apps that I can go to and bring up a message from [church group].

Participant 1, female, 78 years

#### Barriers to Accessing Digital Emotional Well-Being Support

Participants identified several barriers to use of emotional support delivered online, including barriers to using technology in general, such as poor motivation to engage online and difficulties with hearing, vision, or compromised physical ability:

If you ask me if I want online or face to face, I will definitely say face to face ... online, it will be there in my computer, I may not open it...it is easy to put things off if they are online.

Participant 6, male, 73 years

...with the arthritis, now I don’t have the strength in my hand...I use the left hand just to press the buttons.

Participant 4, male, 78 years

Two participants shared a perspective that they would require training and assistance to successfully access online emotional support:

If there was an app that I could put on my phone, then I probably would use it, as long as I knew how to use it. And if I can take it in, but then I need to be shown more than once. That’s my problem. Yeah, it’s learning how to use the damn thing.

Participant 7, female, 70 years

It would need a lot of teaching. I would need a lot of support from my son and my daughter.

Participant 4, male, 78 years

Factors such as relevance, trust, and credibility were also identified by participants as being important for uptake in this cohort:

It has to have a purpose that can be seen and understood.

Participant 4, male, age 78

Yeah, has to be trust around it. So I don’t. I don’t know whether the government can do that ... evaluate those things [digital tools] and give a tick

Participant 6, male, 73 years

#### Support Preferences

Participants were also asked their views on receiving online emotional support. The majority (n=7) of participants expressed a preference for face-to-face support, which was seen as “more personal” (Participant 6, male, 73 years). Despite this, many (n=6) were open to videoconferencing and other digital approaches. Participants expressed that digital approaches could make it easier to access someone to talk to when needed and could also help by providing them with guidance in managing their well-being:

Actually, it’s very good, very good, because patients like us need some help...I can walk and I can do some things, but there are some people that can’t do anything, so it’s better. It’s better to have [digital] services...Definitely convenient to people.

Participant 6, male, 73 years

...To get some ideas of what I can do... to use it and talk to someone, I suppose.

Participant 5, female, 83 years

Well, I guess for people, if they’re feeling a bit down and that...yeah, that they can, you know, look at or to support them. I mean there must be a lot of lonely people out in the world.

Participant 4, male, 78 years

Online emotional support was also seen as potentially appealing to people who were uncomfortable with face-to-face support, as some “don’t like face-to-face” [Participant 3, male, 83 years]. Perceived benefits of digital approaches included providing a sense of anonymity and making it easier to establish rapport or access relevant lived or professional experience:

Maybe if it was online and I don’t see who it is, maybe you could express yourself better. Sometimes I can see people I don’t like. I don’t feel comfortable with them... you’re not going to express yourself if you don't feel comfortable with somebody.

Participant 2, female, 79 years

There were mixed views on accessing virtual emotional support provided through their aged care provider, whether using online programs or apps or support via telehealth. Four participants responded they would welcome this, while 5 reported they might be open to receiving this support from their provider:

Yeah, definitely. I will use it if I get some service from [aged care provider] or from somebody else I will use it...definitely, I will use it.

Participant 6, male, 73 years

If you can just say “I feel depressed,” I can log into [service provider] and say, “I'm a bit down. Can I talk to somebody about something?”

Participant 3, male, 83 years

In contrast, 2 care recipients responded negatively to this proposal, with one person perceiving their care provider as providing physical support only, while the other did not welcome emotional support that was provided outside of their family.

## Discussion

### Principal Findings

This study extends existing literature on the use of digital technologies in later life by focusing on a clinically complex population that has been largely overlooked by researchers but is growing rapidly. Aged in their 70s and 80s, this in-home aged care sample was considerably older than participants in much of the research previously published on this topic, with many researchers defining “older adults” as young as aged 50 or 55 years plus [19,24-26]. Participants in this study generally had limited postschool education, and most reported they were unable to do things others their age could do. Previous research has demonstrated that advanced older age [27-30], poorer health [29,30], and physical disability [28]—all of which are ubiquitous in the home care setting—have a negative impact on use of technology in daily life.

Our findings indicated that while participants generally had good access to digital devices and internet at home, access did not equate to independent or confident use. Technology use varied considerably: some participants used multiple devices for a broad range of activities and functions throughout their daily lives, while others used devices only for basic communication or were unable to use available devices or functions without support. Aged care providers should routinely assess not only device access, but also digital confidence, skills, support needs, and the availability of informal assistance, before introducing digital tools into care.

Most participants used their devices for communication, entertainment, social media, or to access information, while only a minority described using technology to manage their physical health, for example, to monitor their health or to seek health-related information. Participants did not use purpose-designed digital mental health or well-being tools and appeared largely unaware of them, despite their widespread availability in the general population [31]. However, many used technology for hobbies, social connection, music, reading, games, and religious content, which may indirectly support well-being [24]. This suggests that digital well-being tools may be more acceptable if embedded into familiar activities and existing care interactions, rather than presented as stand-alone programs. Aged care providers could address the gap in awareness by recommending apps aligned with their clients’ interests that have a strong evidence base, and by incorporating brief digital check-ins via telehealth.

Despite being identified by their aged care provider as likely experiencing poor emotional well-being, only 1 participant was receiving current professional support, although another had previously received counseling, and a further participant had attempted to access support. Most participants were open to receiving professional support, particularly when framed around having someone to talk to, rather than as mental health treatment. This aligns with previous research describing the reticence of in-home aged care recipients to seek mental health care [32] while also suggesting that many accept formal support at key points in their lives if presented in a nonstigmatizing and personally relevant way. Service providers should frame supportive services in terms of helping the person maintain their well-being, engage in valued activities and interests, or implement lifestyle changes.

Most participants expressed a preference for face-to-face or hybrid emotional support rather than fully online, consistent with previous research [19,32]. However, several were open to using telehealth to access mental health specialists, counselors, or people with lived experience, and identified convenience and anonymity, which are important enablers for digital mental health interventions [19]. Participants also recognized the potential for telehealth approaches to address barriers to accessing care, such as limited mobility. Their perceptions may have been shaped by strong current reliance on informal sources of emotional support. Ready access to family or community support may reduce perceived need for digital alternatives and reinforce preferences for personal, face-to-face interactions. This may help explain participants’ preference for in-person support, alongside their openness to digital tools when positioned as complementary to, rather than replacing, existing support networks.

Digital emotional well-being approaches must account for both general barriers to accessing mental health support, such as negative attitudes, low awareness, availability, and accessibility of services [21], as well as digital-specific barriers. In this study, participants identified challenges related to sensory impairments, reduced physical dexterity, low confidence, privacy and scam concerns, and the need for repeated practical instruction. These findings are consistent with previous research showing that older people may require accessible, reliable, and easy-to-use technologies, supported by clear instructions [11,19]. The current findings indicate that training for home care recipients should be ongoing, rather than one-off, with opportunities for repetition and practical demonstration. Approaches such as in-home coaching, simple step-by-step guides, and embedding support within routine care interactions may help to build confidence and sustain engagement over time.

Trusted health care providers may be well placed to introduce suitable digital well-being tools, either by sharing information about available options or embedding selected tools into service delivery [19,26]. Some participants were enthusiastic about the potential to receive emotional support from their aged care provider, while the majority indicated they might be willing to consider this approach, consistent with other recent research [32]. Future research should identify which types of digital supports are most acceptable and feasible in home-based aged care. While many digital mental health offerings focus on managing symptoms of depression and anxiety, in these interviews, care recipients described a broad range of activities that they saw as helpful for their emotional well-being, which offers the possibility of extending the types of interventions for greater engagement and uptake.

Implementation should address older people’s concerns about making a mistake or damaging technology, privacy, scams, and the credibility of digital tools, all of which may impact their willingness to engage [10,11,20,26]. Most participants in this study required at least some support in using digital technologies at home, typically provided by their adult children. While some only sought assistance after attempting to resolve technology problems independently, family members routinely completed digital tasks on behalf of other older people. Although family support may often be appropriate during implementation [11,33], reliance on family alone may reinforce dependence and limit autonomy. Encouragingly, even among care recipients who reported poor skills in using technology, many were willing to learn, consistent with previous research [33]. Proactive, no-cost support is likely important for successful implementation, given this sample’s poor uptake of existing technological support services available in the community and previous reports of limited use of technology helpdesks [34]. Roles such as digital navigators, technology mediation workers or facilitators within aged care services could support older people’s adoption and use of technology [25,26]. Policymakers should ensure that aged care funding enables older people to access not only apps, devices, and internet services where required, but also the practical support needed to use them. Greater coordination between aged care providers, health services, and community organizations (eg, community-based digital literacy programs) may also help ensure older adults can access consistent and trusted sources of assistance.

The findings can be understood through the lens of the Unified Theory of Acceptance and Use of Technology (UTAUT) [35], which proposes that uptake is shaped by performance expectancy (perceived usefulness), effort expectancy (perceived ease of use), social influence, and facilitating conditions. Participants’ emphasis on technologies needing a clear and meaningful purpose aligns with performance expectancy, with care recipients being more open to using digital tools when they could see how these would support their daily lives or well-being, or help them engage in valued activities. However, low confidence, fear of making mistakes, and the need for repeated instruction reflect low effort expectancy, highlighting perceived complexity as a key barrier.

Social influence played a central role in shaping technology use, with family members, particularly adult children, playing a central role in introducing and supporting technology use, although in some cases they completed digital tasks on behalf of participants. Technology adoption in this population is not solely an individual process but is strongly embedded within social relationships, which may both facilitate and inadvertently limit independent use. Facilitating conditions were often limited, with most participants relying primarily on informal support networks and reporting limited engagement with structured sources of technological assistance for training and ongoing assistance.

Collectively, applying UTAUT highlights that limited uptake of digital technologies to support emotional well-being in home-based aged care is not primarily driven by lack of interest, but by a combination of modifiable factors related to usability, support, and perceived value, as well as lack of awareness of relevant tools. Technology adoption in this population is strongly shaped by reliance on informal carers and the need for ongoing, hands-on support, rather than access alone. Even when interest is high, low effort expectancy and limited support can constrain engagement. Together, this suggests that improving uptake of digital well-being interventions in this population will require not only demonstrating value but also reducing complexity and embedding sustained support within service models. This study extends existing literature on UTAUT by demonstrating how these constructs operate in a clinically complex, home-based aged care population, where reliance on informal carers and physical limitations may amplify effort expectations and limited access to resources.

### Limitations

While this is the first in-depth exploration of the views of in-home aged care recipients in using technology to support their emotional well-being, there are several limitations of this study, most notably the small size of the sample and the potential for sampling bias; those with very low interest in using technology and those uncomfortable discussing emotional well-being may have chosen not to participate in the study. In addition, the study recruited participants considered by their care workers to have poor emotional well-being, given the broad focus of the research. Some of the participants may have experienced symptoms of depression, anxiety, or other mental health conditions, but their mental health status was not formally assessed, and different results may have been obtained with a sample with confirmed mental health conditions. It is also possible, given the absence of formal criteria provided to referring staff, that some participants did not experience poor emotional well-being at the time of the interview; however, all participants referred to either current or previous periods where they struggled with low mood or aspects of poor well-being during interviews. Aged care recipients from regional or rural areas, those who were non–English-speaking, and those with significant cognitive impairment were not included in this study; additional research is required to understand the perspectives of these populations. Similarly, the research was conducted with recipients of a single aged care provider, and the results may not generalize to the broader population of older people who receive aged care at home. Three participants had a family carer present during their interview, which may have influenced how they responded to interview questions. Finally, the interviews were conducted from October to December 2023 and explored technologies readily available at that time. Novel AI-based tools to support emotional well-being, such as chatbots, were not discussed in interviews. With technology evolving rapidly, research findings should be updated regularly.

### Conclusions

Despite the limitations, this study highlights an openness to both formal and informal support for well-being among in-home aged care recipients. Digital technologies could play an important role in supporting a population facing increasing mental health rates [36] and poor access to professional mental health services [7]. As well as increasing access to support, digital technologies have the potential to allow older people to self-manage their well-being. However, our findings suggest that access to everyday digital devices does not necessarily translate into awareness or use of purpose-designed digital well-being tools among frail and clinically complex older people living at home. Aged care providers could play a valuable role in introducing suitable programs to older people, leveraging established trusting relationships and should consider integrating digital tools into existing service delivery to support their clients’ well-being [11].

When considering digital initiatives, aged care providers should prioritize those that can be delivered using the devices older people already access in their homes, particularly smartphones, tablets, and smart TVs. Importantly, many care recipients will require significant and ongoing technology training and support in using new technologies, even those accessed via their existing devices, which requires appropriate resourcing [11]. This study identified several barriers that should be addressed throughout implementation and highlighted the need for a highly tailored implementation approach, given the heterogeneity in care recipients’ digital literacy and experience using technology—one size will not fit all. The routine use of brief assessments of care recipients’ technological readiness has been proposed previously for the aged setting [10]. It may also be beneficial to provide education to aged care workers [10], some of whom may hold negative perceptions about the use of technology in aged care [11,25] and may themselves have low digital readiness [10,11,37], which could impact their willingness to offer these solutions.

## Appendix

Multimedia Appendix 1 Interview schedule.

Multimedia Appendix 2 Results summary table.

## Abbreviations

e-book: electronic book
GP: general practitioner
UTAUT: Unified Theory of Acceptance and Use of Technology

## Data Availability

The qualitative data generated and analyzed during this study are not publicly available because participants did not consent to the sharing of their interview data. Relevant supporting information is provided within the article and multimedia appendices.
