# Supplementary material for: The Use of Digital Technology to Support the Emotional Well-Being of Older People Who Receive Care at Home From the Perspective of Care Recipients: Qualitative Study
Source: JMIR Hum Factors. 2026 Oct 28;13:e93823. doi: 10.2196/93823 (PMC13755775; doi:10.2196/93823)
Supplement: Supplementary file 1 [file humanfactors_v13i1e93823_app1.docx]

## Appendix 1 - Interview Schedule

1. Complete the demographic information form with the interviewee
   a. What year were you born? [enter year]
   b. What is your gender?
      - Male
      - Female
      - Non-binary
      - Other (specify)
      - Prefer not to disclose
   c. What is your country of birth? [enter country name]
   d. What is your highest level of education?
      - Finished postgraduate university
      - Finished undergraduate university
      - Finished TAFE
      - Finished school (year 12 or equivalent)
      - Other (specify)
      - Prefer not to disclose
   e. What is your relationship status?
      - Married
      - De-facto relationship/unmarried co-habitation
      - Never married
      - Widowed
      - Divorced
      - Separated
      - Other (specify)
      - Prefer not to disclose
   f. What is your current work status?
      - Employed, full time
      - Employed, part time
      - Employed, casual
      - Retired
      - Unemployed
      - Volunteering
      - Other (specify)
      - Prefer not to disclose
   g. Do you have health problem that prevents you from doing any of the things people your age normally can do?
      - Yes
      - No
      - Don’t know
      - Prefer not to disclose
2. Begin the interview.  Remind the interviewee the interview is being recorded

*[Thank you for agreeing to take part in this project to help us investigate how technology can support older people to have a happier life. The purpose of our meeting today is for me to understand how you use technology, such as computers or your mobile phone, and to ask your opinion on how you think technology could be used to support older people to maintain a good quality of life and enhance their mood. We understand that older people have a range of different perspectives on the use of technology, particularly for health purposes, and so I would really like to hear what you think. There are no right or wrong answers, I would just like to hear your thoughts and opinions.]*

[Do you have any questions before we begin?]

3. You are receiving care from an aged care provider - what does this currently include?
4. This project explores the opportunity to provide support for people’s emotional wellbeing online. What do you think about this? Do you receive any such services, either online or in person?
5. What do you do to improve your mood or emotional wellbeing?
6. Could you imagine receiving specific support for your emotional wellbeing from your aged care provider? If yes, what would this include? What would be a preferred outcome of this service?
7. What technologies are you currently using? Do you have apps on your phone/tablet/laptop (e.g., YouTube, Netflix, WhatsApp, Facebook, communication)? What do you use those for? How often do you use them?
8. Do you use any technologies to support your mood?
9. How do you think technology could support your mood or emotional wellbeing? Could you imagine yourself using these technologies? What technologies would you like to use to improve your mood or emotional wellbeing? Do you have any concerns about using them?
10. Can you think of anything that could be put on your phone/tablet that might help you with your mood or emotional wellbeing?
11. What do you see as the benefits of an emotional wellbeing app? What do you see as challenges? Would you need support with this? What kind of support?  Who could provide this support?
12. This project examines the opportunity to provide emotional support online. What do you think about this? What would you call a good outcome / result of an online service to support your mood or emotional wellbeing?  Would you need support with this? What kind of support? Who could provide this support?
