# Supplementary material for: The Use of Digital Technology to Support the Emotional Well-Being of Older People Who Receive Care at Home From the Perspective of Care Recipients: Qualitative Study
Source: JMIR Hum Factors. 2026 Oct 28;13:e93823. doi: 10.2196/93823 (PMC13755775; doi:10.2196/93823)
Supplement: Supplementary file 2 [file humanfactors_v13i1e93823_app2.docx]

## Appendix 2 – Results Summary Table

| Theme | Subtheme | Description and frequency | Example Quotes |
| --- | --- | --- | --- |
| Technology Ownership and Use | Technology Ownership | Participants described owning a range of technology, the most common being smartphones (n=11), tablets (n=9) and computers (n=9). | *Got a laptop, iPad. iPhone. So I use all those.* [Participant #5, female, age 83] |
|  | Technology Use | Participants described using technology for a range of different purposes, with the most common being communication (calls [n=11], SMS [n=8], video calls [n=7]), entertainment (streaming apps, [n=7]) and social media (Facebook [n=7]). | *I’m a member of a bowling club and we had Zoom meetings there…I’ve got friends in England we FaceTime it, which is quite good. Or Skype, or you’ve got that many different things you could do. But we've FaceTimed a lot.* [Participant #3, male, age 83] |
|  |  | Only 4 participants described using technology to manage physical health issues, including a smartwatch to record steps and heart rate [n=1], search for health advice online [n=1], and make medical appointments [n=2]. | *I know how to read how many steps I’ve done, and what my heart rate is, and supposedly how much [energy] I’ve burned off.* [Participant #7, female, age 70]  *With Google I found a program which may have come out of America. I’m not sure, but it’s for Type 2 diabetes really… you can find programs that show exactly what not to eat.* [Participant #11, female, age 87] |
|  |  | The majority of participants (n=8) reported low to moderate usage of the functionality of the technology that they owned.  Three participants in this group reported having technology in their home that they did not use.  Five participants in this group described difficulties using core functions, such as email and SMS text messaging. | *My daughter does all the emails and everything* *and not that she hasn't got enough to do, but yeah. I can send a message, receive a message, I* *can play games on my phone. I can take photos and that's it. The computer… it's got a square across on it [I don’t use it]* [Participant #7, female, age 70]  *I’ve got an iPad but I don’t use it.* [Participant #8, male, age 86]  *I’m just learning to message. Takes me … sometimes I think it’s quicker to ring than for me to message.* [Participant #9, female, age 75], |
|  |  | Some participants (n=3) described high technology use, making use of a broad range of functionality across multiple devices. | *I just put it on in the morning and I say jazz, Alexa and just have music all day* [Participant #11, female, age 87] |
|  | Support in Using Technology | Some participants (n=4) reported confidence in using multiple technologies and positive attitudes toward using and learning new technologies. | *…[I use] e-mail, and call people and text. And I'm using the, for the international [calls], I use WhatsApp. It's great for international calls, but I know I phone all my family. [Interviewer: do you use FaceTime and things like that?] Yeah, FaceTime is in with the Apple one, so I don't now I don't have Apple phone or Apple devices…I like my phone android*  [Participant #6, male, age 73]*It’s just interesting, it’s a new challenge. Something nice, new to learn, yeah.* [Participant #11, female, age 87] |
|  |  | The majority (n=8) of participants described receiving support around technology from family, often adult children to either complete the tasks for them or for support. | *The daughter has on her computer because, um, like I got…a message I guess it was saying that the registration was due on the car and so I took it around and she said ‘there you are, done and paid for’ because she can have access to my bank account.* [Participant #4, male, age 78]  *Sometimes I'll forget a couple of passwords, then I’ll ring my son, and I'll say ‘what's the password?’* [Participant #5, female, age 83] |
|  |  | Adult children were the primary source of training and support, however one participant did report receiving support from their local community. | *I've got them all on there [apps on my TV], every one of them. The kids put everything on there* [Participant #11, female, age 87] |
|  | Barriers to Technology Use | Five participants expressed concerns related to privacy and security, in part due to experiencing spam calls and messages. | *I do worry with what they talk about, about the spams and everything, and I've had a few phone calls....I'd rather withdraw all my money out of the bank and have it here than go online. I don't purchase anything online…* *People with brains get ripped off, let alone somebody like me, so I won’t do it.* [Participant #7, female, age 70] |
|  |  | Two participants identified cost as a barrier to owning or using technology. | *It all depends on the app actually, how much it costs* [Participant #3, male, age 83] |
|  |  | Two participants identified a concern regarding doing the wrong thing or breaking the technology. | *I’m a one finger person and then it'll take me all day just to write a little paragraph. And if I press the wrong thing. Yeah, that's what I'm worried about.* [Participant #7, female, age 70] |
|  |  | Three participants identified adjustments they needed to make to their technology to increase usability (e.g. increase font size). | *Yes (I use it) but I don't like the phone because it's too small. I can't read and every, every time I have to make [the font] bigger, bigger and read, so it's big.* [Participant #6, male, age 73] |
| Support for Emotional Wellbeing | Current Sources of Support | The most common source of emotional support was from family (n=7). | *I got it from my family members…I like to talk about myself with my family members, not to others* [Participant #6, male, age 73]  *I feel more calm and more comfortable talking to family members.* [Participant #2, female, age 79] |
|  |  | The second common source was through the community, including friends and neighbors (n=6). | *“[My prayer group have] all been really, really great supporters… I could ring them anytime, day or night…and I could talk to them…That helped me a lot.”* [Participant #10, female, age 79]  *Well, if I needed support then I've got my daughter. I've got friends in the village here.*  [Participant #5, female, age 83] |
|  |  | Professional support was only described by two participants, though one of these participants was describing historical support. | *I’ve got another 4 visits, but it’s up to the doctor, I think to ask for more if I need it. As soon as I mentioned it, he was straight on [organized a referral]. I had [a phone call] the next day.* [Participant #7, female, age 70]  *I used to [be depressed], but not anymore…oh, going back how many years? 1995. So, it's been a lot of years and I, I spoke to a counselor and that and yeah, I’m fine.* [Participant #10, female, age 79] |
|  | Barriers to Seeking Support | Barriers include not wanting to be a burden and preferring to discuss personal details with family. Another cited issues in accessing support that had affected him personally. | *I’d feel more comfortable with my family member, than talking to a stranger. Yeah, I sort of can talk better to my family and that way… I think I can get a lot more off my chest by talking to family.* [Participant #2, female, age 79]*I must admit everything seems to just take so long, and, and you get to the point where you just say ‘Oh, forget it!’ And you don't carry on with it.* [Participant #8, male, age 86] |
|  | Willingness to Engage in Support | The majority of participants (n=10) indicated that they were open to receiving support. However narratives showed initial reticence, that shifted to acceptance as they explained their perspective. | *No, I don't really because to me it's all new. I’d, I've never considered it because I've never thought that I would use anything like that. I mean, there's nothing there to use at the moment, I agree, but it was the same as going to the pub…Yeah, I just didn't think it was there, and so I never thought about it.* [Participant #8, male, age 86] |
|  |  | The majority (n=10) preferred professional support, with one participant indicating a peer-support model would be preferable. | *If I was able to be in contact with somebody [at Silverchain] to talk when I’m really down…that would be good* [Participant #7, female, age 70] |
|  |  | The one participant who did not wish to engage in emotional support cited speaking with strangers as the barrier to engagement. | No, no. Strangers. They'd be strangers. People I didn't know that would be talking, probably from rote. No, no, it wouldn't, wouldn't mean a thing to me. [Participant #11, female, age 87] |
| Use of Digital Technologies to Support Emotional Wellbeing | Current Use of Technology to Support Emotional Wellbeing | Seven participants reported using technology to support their emotional wellbeing through music, hobbies, reading, and remote religious support. | *Yes, like with YouTube at the moment. Russell Coight. I don't know whether you’ve ever seen him on TV an Aussie Guy. There was an Outback adventures show with Russell Coight and if anything can go wrong, it happens with him. And yeah, I get a laugh out of those cause I used to watch his show when he was on TV.* [Participant #4, male, age 78] |
|  | Barriers to Accessing Digital Emotional Wellbeing Support | Participants identified a range of barriers to using technology itself, from low motivation to physical issues such as dexterity, and low hearing or vision. | *I can’t hear properly even on the phone sometimes. If they talk too fast, or…the voices are too sharp…I can’t hear it.* [Participant #11, female, age 87] |
|  |  | A few (n=2) identified that there would be training needs for any online emotional support. | *It would need a lot of teaching. I would need a lot of support from my son and my daughter* [Participant #4, male, age 78] |
|  |  | Participants identified trust, relevance and credibility as critical factors which must be addressed for successful uptake in this cohort. | *Yeah, has to be trust around it. So I don’t. I don’t know whether the government can do that … evaluate those things [digital tools] and give a tick [Participant #6, male, age 73]* |
|  | Support Preferences | Over half of participants (n=7) expressed a preference for face-to-face support. However, many (n=6) were open to digital approaches. | *It would be better face to face…unless you’re doing it through FaceTime…that would be fine.* [Participant #5, female, age 83] |
|  |  | Participants identified particular groups of older people who may prefer online support. | *Face to face is good, but some people don’t like face to face. [Participant #3, male, age 83]* |
|  |  | Participants identified benefits of taking part in online discussions including a sense of anonymity. | *Maybe if it was online and I don't see who it is, maybe you could express yourself better. Sometimes I can see people I don't like. I don't feel comfortable with them. So you, you're not going to express yourself if you don't feel comfortable with somebody.* [Participant #2, female, age 79] |
|  |  | Views were mixed on accessing emotional support through their aged care provider, with 4 indicating it would be welcome and 5 indicating they might be open to this service. Two participants did not welcome emotional support as a service through their aged care provider. | *First of all I need to learn how to use the computer. Yeah. And then like if I was able to be in contact with somebody to talk when I'm really down, yeah. Yeah, that would be good.* [Participant #7, female, age 70] |
